# A Qualitative Study of the Views of Ethnic Minority Healthcare Workers Towards COVID-19 Vaccine Education (CoVE) to Support Vaccine Promotion and Uptake

**DOI:** 10.1177/10482911241273914

**Published:** 2024-09-17

**Authors:** Holly Blake, Vinishaa Premakumar, Abishaa Premakumar, Aaron Fecowycz, Sala Kamkosi Khulumula, Wendy Jones, Sarah Somerset

**Affiliations:** 1 School of Health Sciences, 6123 University of Nottingham, Nottingham, UK of Great Britain and Northern Ireland; 2 NIHR Nottingham Biomedical Research Centre, Nottingham, UK of Great Britain and Northern Ireland; 3 School of Medicine, 6123 University of Nottingham, Nottingham, UK of Great Britain and Northern Ireland; 4 BAME Shared Governance, 9820 Nottingham University Hospitals NHS Trust, Nottingham, UK of Great Britain and Northern Ireland; 5 Occupational Health Consultant, Nottingham, UK of Great Britain and Northern Ireland

**Keywords:** vaccine, COVID-19, ethnic minority, workforce, digital, health education

## Abstract

Ethnic minority healthcare workers (EMHCW) are at high risk of COVID-19 infection and adverse health outcomes, but vaccine uptake is low among ethnic minority communities, including EMHCW. We explored the views of EMHCW towards COVID-19 Vaccine Education (CoVE), a digital training resource to improve knowledge and confidence for promoting the COVID-19 vaccine. Thirty EMHCW completed CoVE, then participated in a semi-structured qualitative interview. Principles of framework analysis were used to deductively analyse data using concepts from the Kirkpatrick New World Model of training evaluation. CoVE was viewed to be engaging, accessible and relevant to EMHCW. This training increased EMHCW perceived knowledge and confidence to provide evidence-based information to others, dispel myths, and reduce vaccine hesitancy. Participants reported changes in vaccine promotion behaviours and vaccine uptake. CoVE could be used to help improve vaccine literacy among EMHCW, enhance health communications about vaccines, and ultimately help facilitate uptake of occupational vaccination programs.

## Background

Despite the introduction of safe, effective vaccines, severe acute respiratory syndrome coronavirus 2 (SARS-CoV-2) continues to mutate and spread across the world.^1^ As of 31 March 2024, more than 774 million confirmed cases and more than 7 million deaths from COVID-19 have been reported globally.^1^ Vaccines remain the cornerstone of actions to prevent serious illness and deaths from COVID-19.^2^ The UK Coronavirus Dashboard showed that, by August 2023, more than 151 million doses of vaccine had been administered in England.^3^

Although willingness to be vaccinated is generally high across the UK (>80%, *n* = 12,035),^4^ data from the Office for National Statistics (ONS) show variations in vaccine uptake rates with more unvaccinated adults in certain population subgroups, such as those who identify as Black, Caribbean, Black African, or White Other, those living in deprived areas, those who have never worked or are long-term unemployed, those who are limited a lot by disability, who are male, or who identify as Muslim or as having ‘Other Religion’ (for England and Wales, the religious groups with ONS data available are: No religion, Christian, Buddhist, Hindu, Jewish, Muslim, Sikh, Any other religion).^5^ Similarly, research shows vaccine hesitancy exists in population sub-groups (i.e., women and those of younger age) or in those with specific factors (i.e., lower educational level), and there are high levels of vaccine hesitancy in certain ethnic minority groups.^4^ Vaccine hesitancy has been identified by the World Health Organization as 1 of the 10 leading threats to global health. According to the SAGE Working Group, it is referred to as a delay in acceptance or refusal of vaccination despite availability of vaccination services.^6^ Influencers of vaccine hesitancy are time- and context-specific,^7^ but other key factors include lower income level, exposure to misinformation, a perception that COVID-19 is low risk, concerns about vaccine safety and efficacy (including boosters), and a mistrust of government and public health authorities.^2^ In ethnic minorities, vaccine hesitancy is also associated with structural and systemic inequities.^8^ Combined with a narrative around ‘pandemic fatigue’^9^ and the general waning of public attention to preventive behavioural strategies (e.g., ventilation, face coverings, handwashing, social distancing), vaccine hesitancy presents a significant challenge to the success of health protection programs, including those delivered in occupational settings.

As the most trusted source of vaccine-related information, healthcare workers (HCW) have a powerful influence over other people's vaccination decisions.^10^ The public is more *willing* to receive a vaccine if a healthcare provider recommends it,^11^ and early research showed that vaccination *rates* can be higher when HCW are positive about vaccines.^12^ While advocating vaccination uptake is key to HCW roles, COVID-19 vaccine hesitancy exists among HCW themselves. UK Health Security Agency (UKHSA) data for England^13^ show that only 36.3% of frontline HCW with direct patient contact received a COVID-19 vaccine between 1 September 2022 and 30 November 2022. This is lower than the uptake for seasonal influenza vaccination during the same period at 41.8%. There are occupational differences in vaccine uptake, with lower uptake among nurses and support staff compared to doctors and other professionally qualified clinical staff.^13–15^

There are myriad reasons for engaging in efforts to increase the uptake of EMHCW in occupational health vaccination programs. First, low uptake of EMHCW in occupational health vaccination programs has implications for vaccination decisions of other EMHCW (as well as the success of community vaccine programs). That is, vaccinated HCW are more likely to recommend vaccination to others,^10^ and it has been proposed that unvaccinated HCW put themselves and others at increased risk of illness.^16^ HCW have a greater exposure to COVID-19 and at higher risk of infection and death compared to the public. EMHCW are particularly at risk since their work exposes them to infection, and there are known ethnic inequalities in COVID-19 infections, intensive care unit admissions, and death. Third, an unvaccinated workforce may present a burden to healthcare employers since unvaccinated HCW report more symptoms and have longer sick leave duration than fully vaccinated HCW.^17^ The dramatic increases in healthcare workforce staff absence due to COVID-related reasons^18^ and associated ‘workforce loss’ due to COVID-19 sickness absence and death across all occupational groups^19^ adds pressure to already burdened healthcare services in which healthcare demand is outstripping workforce capacity.^20^ Vaccinating HCW therefore remains a global priority.^21^

While uptake rates suggest that some HCW are personally hesitant about vaccination,^13^ others may simply feel ill-equipped to recommend vaccination or have conversations to explore concerns in those who are reluctant or refuse vaccination.^10^ A review (*n* = 185 articles) showed that awareness and knowledge about vaccines have increased HCW confidence in and willingness to recommend a vaccine.^10^ HCW should therefore have access to appropriate training and education related to the benefits and risks of COVID-19 vaccines, with the aim of encouraging them to attend for vaccination themselves (e.g., through occupational health vaccination offers) while providing them with the knowledge and confidence to proactively advocate vaccine uptake in others.

The use of digital resources offers flexibility in the delivery of HCW education,^22^ with online learning likely to increase beyond the pandemic.^23^ One example is an e-health intervention called COVID-19 Vaccine Education (CoVE) developed by Blake and colleagues in 2022 to facilitate global promotion and uptake of the COVID-19 vaccines.^23, 24^ CoVE is an interactive, multimedia training resource for HCW that aims to increase knowledge about the individual and societal benefits of COVID-19 vaccination, as well as to help HCW address reasons for vaccine hesitancy, dispel myths, and improve vaccine literacy.^24^ During the development process for CoVE,^24^ 48 HCWs (*n* = 28) and trainees (*n* = 20) were involved in group consultations to establish the aims. Due to variations in vaccine hesitancy and uptake by ethnicity in the UK,^5, 25^ ethnic minority HCW and trainees were purposely over-sampled (35% and 65% ethnic minority participants, respectively).^24^ Within this group, 9 were vaccine hesitant, of which 8 were from ethnic minority communities. The peer review panel involved in content development was from 7 countries (United Kingdom, United States of America, Pakistan, Jordan, Turkey, Thailand, and Malawi). For the evaluation of CoVE with HCW,^24^ 14 interviews were completed by participants from 5 nationalities (British, Filipino, Polish, Lebanese and Pakistani), and a survey was completed by 162 participants from 26 countries (Algeria, Australia, England, Finland, Ghana, Greece, Guernsey, France, Ireland, India, Indonesia, Italy, Jordan, Lebanon, Malawi, Nigeria, Pakistan, Philippines, Poland, Romania, Scotland, South Africa, Thailand, Uganda, United States and Wales). Although ethnicity was not reported, the original evaluation therefore included users from diverse cultural groups and geographic regions. CoVE training, when made publicly available online during the COVID-19 pandemic, increased HCW self-reported knowledge and confidence relating to vaccine promotion and was perceived to facilitate vaccine-promoting behaviours and vaccine uptake in HCW from 23 countries.^24^

However, the engagement with and perceived usefulness of CoVE to EMHCW in the UK are unclear. Health literacy, barriers, and enablers to communication with others about COVID-19 vaccines, approaches to encouraging vaccine uptake, and the perceived value and relevance of CoVE training may vary with ethnicity, context, and setting. In the context of high SARS-CoV-2 infection rates at the time of writing,^1^ targeted efforts were urgently needed to address low vaccine uptake and high vaccine hesitancy in EMHCW. As trusted advisors, EMHCW are well positioned to contribute to improving vaccine uptake not only in patients and the public, but also among their colleagues. Addressing these research gaps may also inform occupational health policy by supporting and enhancing vaccine literacy in EMHCW, and second, encouraging vaccine uptake in EMHCW, particularly those involved in workforce vaccination programs.

The aim of the study was to explore whether CoVE training improves EMHCW knowledge and confidence for promoting the COVID-19 vaccine and/or leads to changes in behaviour around vaccine promotion and uptake.

## Methods

**Figure 1.**
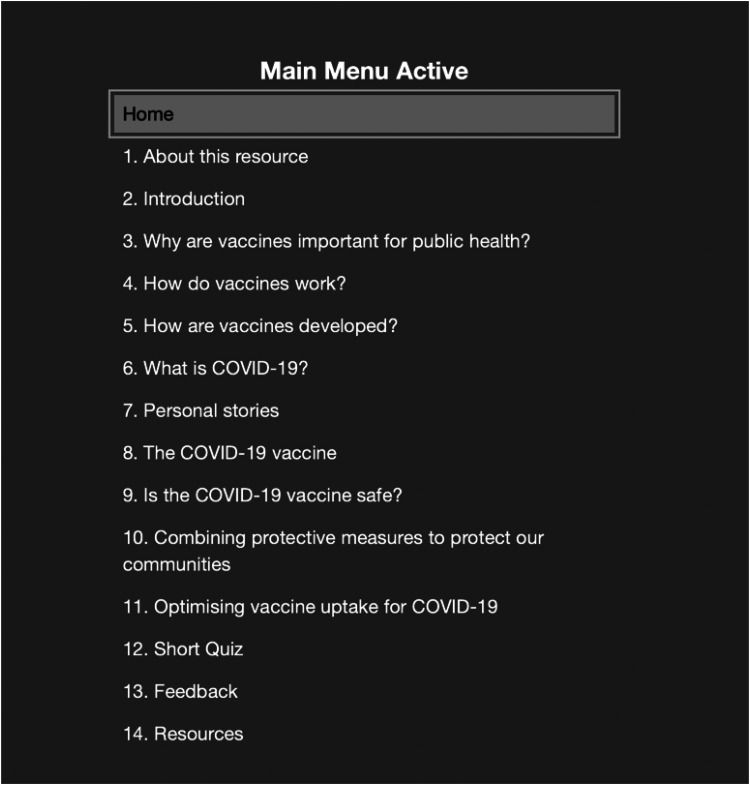
COVID-19 vaccine education (CoVE) Version 2 content summary.

**Figure 2.**
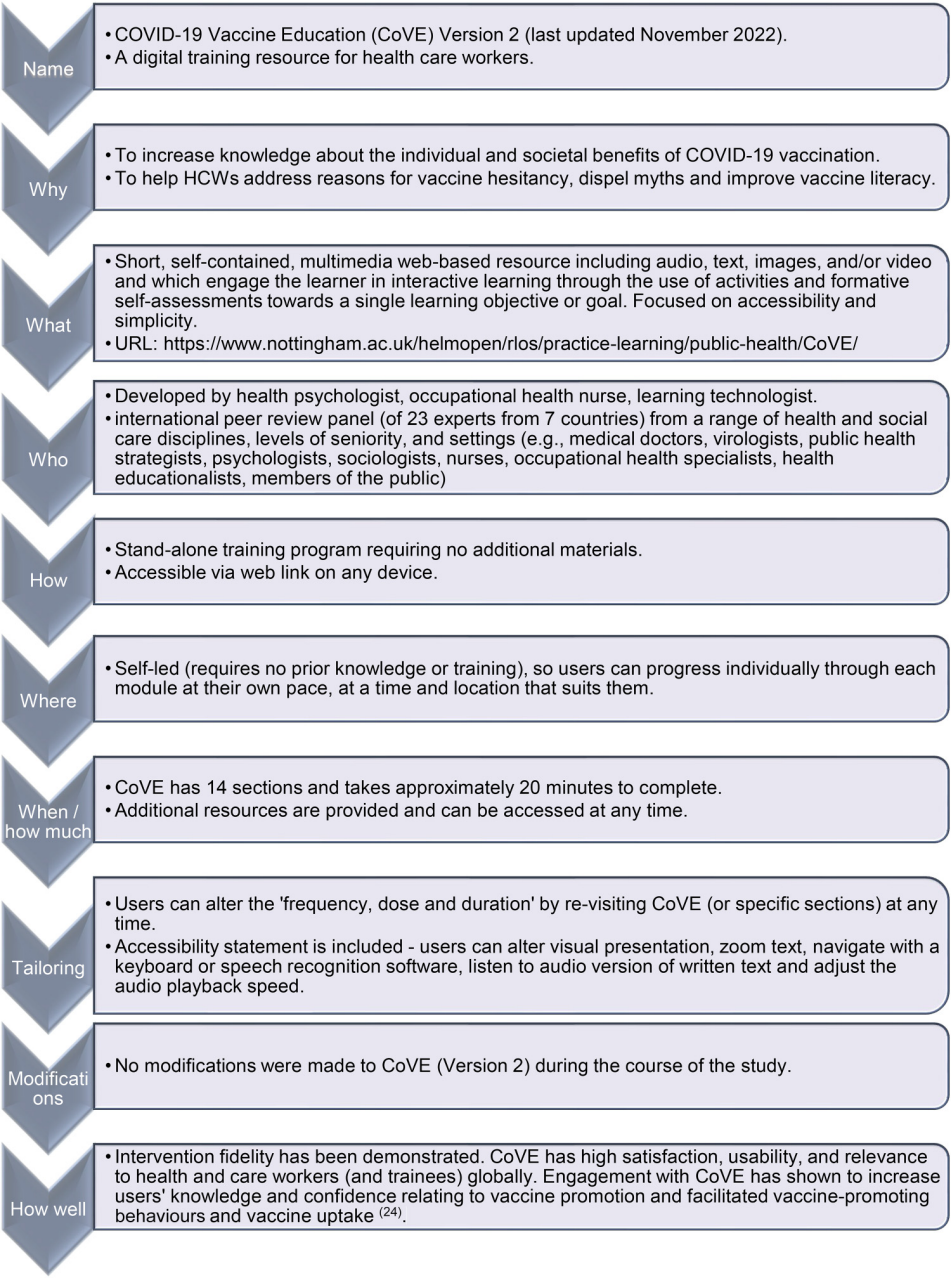
TIDieR description of COVID-19 vaccine education.

**Figure 3.**
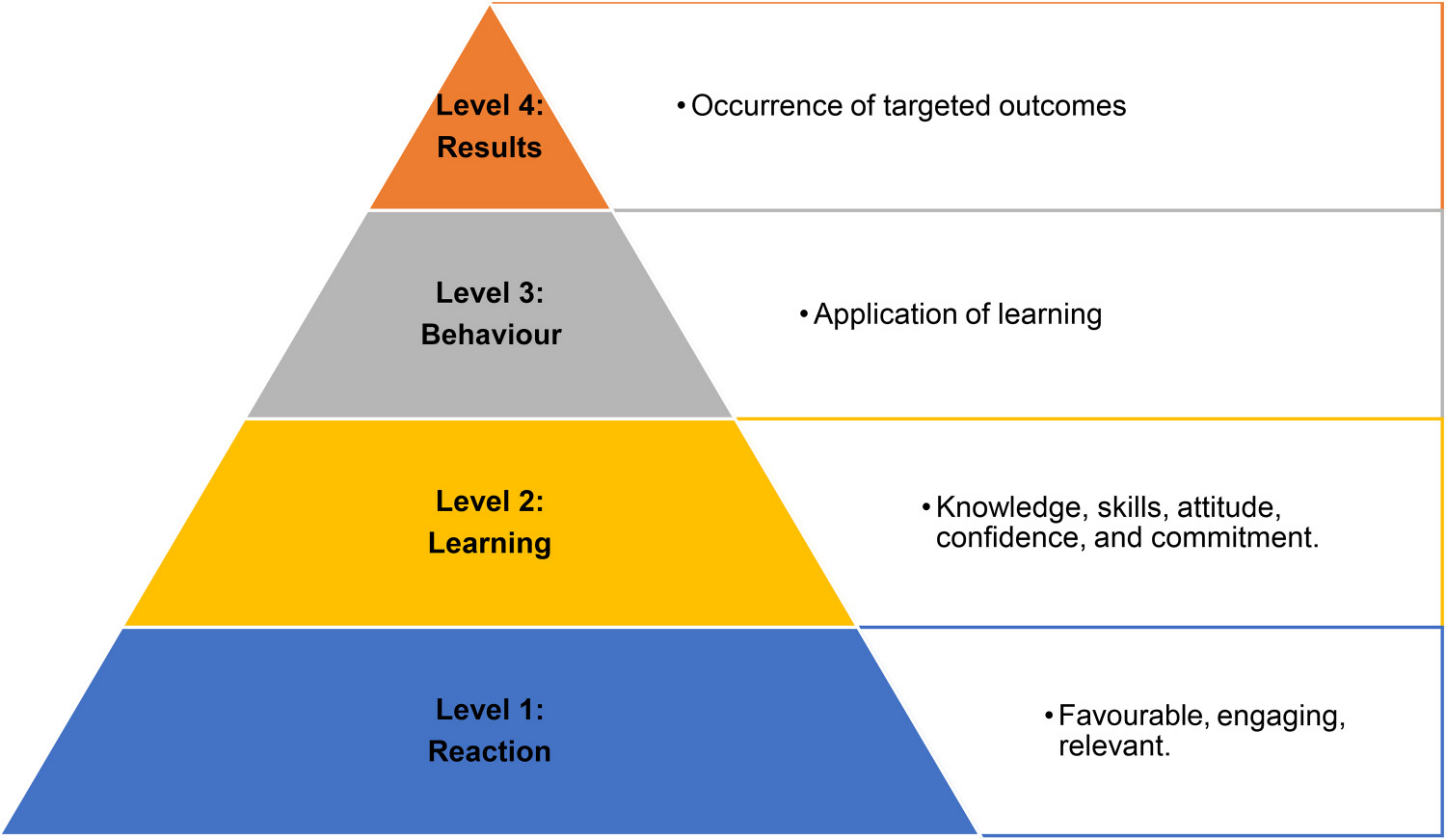
Kirkpatrick's four levels of evaluation.

We conducted a single-group intervention study with a qualitative evaluation of EMHC. The intervention was CoVE, a digital training package developed for health and care workers.^23^ Figure 1 provides a summary of CoVE content, while Figure 2 displays the Template for Intervention Description and Replication (TIDieR) checklist and guide.^26^ The Kirkpatrick New World Model for training evaluation^27^ (Figure 3) provided the framework for this study and the approach to data collection and analysis. Reporting is guided by the Consolidated Criteria for Reporting Qualitative Research (COREQ-32).^28^ Approval to conduct the study was received from the University of Nottingham Faculty of Medicine and Health Sciences Research Ethics Committee (REC ref. no. FMHS 310-0721).

### Study Population

Eligible participants were EMHCW working in healthcare organisations in the UK. We defined ethnic minority’ (referred to in the UK as all ethnic groups, except White British) to include ‘minority ethnic’, ‘minoritised ethnic’, ‘Black and Minority Ethnic (BME)’, ‘Black, Asian and minority ethnic (BAME)’ or ‘global majority’.

### Recruitment/Sampling of Participants

Purposive maximum variation sampling was initially adopted. To capture a wide range of perspectives, we aimed to recruit participants who were health or care workers (in any UK health or care setting) identifying as being from an ethnic minority background, reflecting diversity in age, gender, occupation, and level of seniority (reflected here by having line management responsibility, or not). Female participants were over-sampled to reflect the gender balance in health and social care (70% female across 50 countries^29^). Participants were recruited online via advertisements circulated via professional networks and postings on social media (eg, X, formerly known as Twitter). Since the study was conducted during the COVID-19 pandemic, recruitment of HCW was challenging due to competing demands on HCWs’ time; therefore, an additional strategy, snowball sampling, was adopted. That is, research participants helped to identify other potential participants who were then recruited via direct approach from the research team, either by email, or face-to-face (i.e., if potential participants requested to speak to the research team following a presentation about the study plans at an EMHCW network meeting).

### Data Collection Methods

All participants received a study information sheet, provided online consent, completed CoVE training online and participated in a post-training qualitative semi-structured interview, within 6 weeks of training completion. Due to rolling recruitment, interviews were conducted over a 10-week period between December 2022 and April 2023 by the second author (VP) who was not involved in design or development of CoVE. Recruitment was stopped when thematic saturation^30^ appeared to have been achieved, with no new information being acquired for any level of the Kirkpatrick model.^27^ Interviews took place via video-call and on average lasted 18 min. Only the researcher and participant were present during the interview, the researcher recorded field notes. An interview topic guide was used (Supplementary file S1). The topic guide was based on that used in the qualitative element of the original CoVE evaluation study;^24^ therefore, we took a similar approach by mapping questions to the Kirkpatrick New World Model of training evaluation.^27^ Interviews were recorded with consent and transcribed verbatim. Participants were given the option to participate in a prize draw for a £50 high street shopping voucher.

### Data Analysis

The principles of framework analysis^31^ were used, involving 4 stages: transcription, coding, charting, and interpretation, to deductively analyse data (applying predetermined codes to the data) using concepts from the Kirkpatrick Model.^27^ The process was overseen by an experienced qualitative researcher (SS). Verbatim transcription of interviews was undertaken by the third author (AP), and the fidelity of each transcript was reviewed by the second author (VP). VP is a medical trainee, and AP is a health researcher. Both had undertaken training in qualitative methods. The researchers familiarized themselves with all interviews using the recording, transcript, and the interviewer's contextual or reflexive notes. Coding was undertaken in NVivo 12.0 (QSR International). A code is a descriptive or conceptual label that is assigned to excerpts of raw data.^31^ The analytical framework (Supplementary Table S2) was derived deductively, with 11 codes identified a priori with a focus on Kirkpatrick Level descriptors^27^: Level 1 Reaction (favourable, engaging, relevant), Level 2 Learning (knowledge, skills, attitudes, confidence, commitment), Level 3 Behaviour (application of learning: behaviour changes), and Level 4 Results (occurrence of targeted outcomes). Although the approach was broadly deductive, inductive open coding (identifying any new codes in the data) was undertaken on all transcripts to allow identification of novel or new topics and the addition of new codes to the framework. Three authors independently coded each transcript (VP, AP and SS) with investigator triangulation to maximize coding reliability. A working matrix was generated by charting codes into appropriate themes. Themes are interpretive concepts or propositions that describe or explain aspects of the data. The matrix was reviewed at various stages throughout the analysis by co-authors (SS, HB, WJ and SKK) to ensure the inclusion of different interpretations. Study participants were invited to review the matrix as part of member checking. The final framework consisted of 123 codes, 19 subthemes, and 11 themes.

## Results

A total of 30 participants consented and were interviewed (90% female). Participants (Table 1) were aged between 21 and 60 years (21–30 years: *n* = 20; 31–60 years: *n* = 10), and 5 had line manager responsibilities.

**Table 1.** Participant Characteristics.

ID	Gender	Age	Employment status	Line manager Y/N	Ethnicity	Professional category
**100**	F	41–50	FT	N	Black African/Caribbean/Black British	Admin/clerical
**101**	F	41–50	FT	N	Black African/Caribbean/Black British	Registered nurses/midwives
**102**	F	41–50	FT	N	Black African/Caribbean/Black British	Registered nurses/midwives
**103**	F	41–50	FT	Y	Asian/Asian British	Registered nurses/midwives
**104**	F	41–50	FT	Y	Black African/Caribbean/Black British	Central/corporate functions
**105**	F	51–60	FT	N	Asian/Asian British	Registered nurses/midwives
**106**	F	21–30	FT	N	Black African/Caribbean/Black British	Healthcare support worker
**107**	F	21–30	FT	N	Asian/Asian British	Medical and dental (physician associate)
**108**	F	21–30	FT	N	Black African/Caribbean/Black British	Registered nurses/midwives
**109**	F	41–50	FT	N	Asian/Asian British	Registered nurses/midwives
**110**	F	21–30	FT	Y	Black African/Caribbean/Black British	General management (Ward manager)
**111**	F	51–60	BS	N	Black African/Caribbean/Black British	Registered nurses/midwives
**112**	F	51–60	FT	Y	Asian/Asian British	Admin/clerical
**113**	M	21–30	FT	Y	Asian/Asian British	AHP/healthcare scientists/scientific and technical (pharmacist)
**114**	F	21–30	FT	N	Asian/Asian British	AHP/healthcare scientists/scientific and technical (sonographer)
**115**	F	21–30	FT	N	Asian/Asian British	AHP/healthcare scientists/scientific and technical (pharmacist)
**116**	F	21–30	PT	N	Asian/Asian British	Non-nursing clinical support (vaccinator)
**117**	M	21–30	PT	N	Asian/Asian British	Non-nursing clinical support (vaccinator)
**118**	F	21–30	FT	N	Asian/Asian British	AHP/healthcare scientists/scientific and technical (biomedical scientist)
**119**	F	21–30	FT	N	Asian/Asian British	AHP/healthcare scientists/scientific and technical (pharmacist)
**120**	F	31–40	FT	N	Asian/Asian British	Medical and dental (physician associate)
**121**	M	21–30	FT	N	Asian/Asian British	AHP/healthcare scientists/scientific and technical (biomedical scientist)
**122**	F	21–30	FT	N	Asian/Asian British	AHP/healthcare scientists/scientific and technical (pharmacist)
**123**	F	21–30	PT	N	Asian/Asian British	Non-nursing clinical support (vaccinator)
**124**	F	21–30	FT	N	Asian/Asian British	AHP/healthcare scientists/scientific and technical (optometrist)
**125**	F	21–30	FT	N	Black African/Caribbean/Black British	Medical and dental (physician associate)
**126**	F	21–30	FT	N	Asian/Asian British	Medical and dental (physician associate)
**127**	F	21–30	FT	N	Other ethnic group	Medical and dental (physician associate)
**128**	F	21–30	FT	N	Asian/Asian British	Medical and dental (physician associate)
**129**	F	21–30	PT	N	Asian/Asian British	Non-nursing clinical support (vaccinator)

*Note*. Study participants are referred to in the text by their participant identification (ID) number and gender identification (e.g., ID100F).

Abbreviations: F, female; M, male; AHP, allied health professions; FT, full-time; PT, part-time; BS, bank staff; Other ethnic group, any other ethnicity not listed; Y, yes; N, no.

In alignment with the principles of Level 1 of the Kirkpatrick model, all participants in our study were willing to engage with CoVE and found the resource to be easy to use and offer flexibility as a digital resource that could be accessed anywhere, at any time, on any device. The combination of visual media, audio narrative, and written text within the resource was seen to be engaging and increase accessibility. The interactive elements were seen as an important mechanism for maximizing learning from the resource and facilitating users in checking their level of understanding to consolidate learning. Content was perceived to be relevant to the healthcare workforce, and specifically, EMHCW. Most participants described CoVE content as being relevant to themselves directly (ie, as healthcare professionals making personal choices about being vaccinated) and/or to their job role (ie, to equip them with the skills and knowledge to facilitate the delivery of health communication to others). CoVE was broadly viewed to be a useful tool to facilitate the uptake of occupational health vaccination programs. Those with existing knowledge about the vaccine felt reassured through trust in the evidence-based content, while those with less prior knowledge found their new learning a source of comfort in uncertain times. While CoVE was widely perceived to be relevant for all HCW, many participants viewed CoVE to be particularly valuable to help with addressing the concerns of EMHCW about being vaccinated themselves via occupational health vaccination programs. Many participants reported that the materials were relevant for helping EMHCW to correct misinformation and dispel myths in others, commonly relating to concerns about the development processes for the vaccines, side effects of the vaccines or religious concerns about vaccination.

In alignment with Level 2 of the Kirkpatrick model, our participant sample was pro-vaccination, with diverse prior knowledge about vaccines. CoVE reinforced their positive attitudes and participants trusted the evidence-based information. Most participants reported that they had gained new knowledge from the training; examples shared related to vaccine development and testing processes, and safety of vaccines during pregnancy and breastfeeding. Some participants reported a dramatic increase in their knowledge. Most of the participants believed that CoVE had increased their confidence to engage in conversations with others about vaccination and were committed to implementing the knowledge in practice (ie, vaccine promotion behaviour) and sharing their learning and/or the resource itself. It was widely recognised that vaccine hesitancy was very high in ethnic minority communities including EMHCW colleagues – this was often associated with religious beliefs, potential adverse effects, or uncertainty about long-term outcomes of vaccines. Participants believed that CoVE could help to change people's attitudes about vaccines by reducing fear and stigma around vaccination, and reducing vaccine hesitancy (in themselves, and others). Nonetheless, it was emphasized that the vaccination concerns of ethnic minority communities needed to be listened to, and that vaccination is a personal decision.

In terms of Level 3 of the Kirkpatrick model, since the interviews were conducted within 6 weeks of training completion and took place after the rollout of the 2022 autumn vaccines, there were limited opportunities for participants to change their own behaviours, encourage colleagues to vaccinate, or ‘apply learning on the job’ with patients and the public. Despite this, changes to vaccine promotion practices were commonly reported. Most of the participants had already implemented learning from CoVE to support health communications (taking place within and outside of their job roles), a ‘critical behaviour’, which impacted other people's vaccination decisions. This included initiating conversations about vaccines with co-workers (eg, to encourage uptake of occupational health vaccination offers among their colleagues), as well as patients and their families (eg, to encourage public uptake of other occupational health and/or community vaccination programs). Implementation of CoVE learning was not limited to those with less prior experience or health protection training but included individuals working in more senior roles (ie, line managers) and those already working as trained vaccinators. Beyond vaccination, a few individuals reported that CoVE served as a behavioural ‘nudge’ to re-engage with numerous protective behaviours that had lapsed or were no longer mandatory (eg, awareness of ventilation, face coverings, hand hygiene, social distancing, COVID testing). Many participants had actively shared the resource with other HCW, and EMHCW; some had not done so by the time of interview, but intended to in the future.

The study time frame limited opportunities to attain consistent evidence for Level 4 of the Kirkpatrick model. However, two participants referred to getting vaccinated themselves after using CoVE. Several participants indicated that they had changed other people's minds about the benefits of vaccination, and around one-quarter of the participants indicated that CoVE had led to others getting vaccinated. This was associated with our participants sharing the resource directly with their colleagues or using their learning to support conversations about the vaccine with colleagues, family, friends, patients, and members of the public from ethnic minority communities (Table 2).

**Table 2.** Illustrative Quotations Aligned with Kirkpatrick Model Levels.

Level	Component	Illustrative quotation
Level 1: Reaction	Favourable, engaging, relevant.	Favourable: ‘…reference of where you got the information from, it makes it very credible’. (ID111F)
		Favourable: ‘…it was small. It was quite quick as well. It wasn't… very long …some people can be put off by that’. (ID126F)
		Favourable: ‘more comfortable after I’ve read it’. (ID124F)
		Engaging: ‘…the visual, the voice, the listening. So, you've catered for people with different, different needs’. (ID104F)
		Engaging: ‘…there was a quiz as well at the end and that really helped to test my knowledge’. (ID117M)
		Engaging: ‘I think the interactive elements are really good, especially for me because I'm dyslexic, so sometimes reading all of the information is kind of hard’. (ID107F)
		Engaging: ‘It's just mostly the information that we've got from the videos’. (ID109)
		Relevant: ‘… reading through this package it's, it's, answered a few of the concerns that I had’. (ID104F)
		Relevant: ‘… something like this would really help all of us to get a better understanding of what we're putting inside our bodies and how it benefits us’. (ID114F)
		Relevant: ‘… people think that the COVID vaccine hasn't been tested enough or it's unreliable or it's dangerous. And those are the kind of myths that have been, you know proved wrong by this package’. (ID122F)
		Relevant: ‘could be emailed for example from like GPs or hospital staff members to patients. Definitely get it out to all healthcare professionals out there because it's very useful’. (ID115)
Level 2: Learning	Attitudes, knowledge, skills, confidence, commitment.	Attitudes: ‘… within our community group…there's a lot of reluctant to have the vaccine’. (ID103F)
		Attitudes: ‘I think stigma normally starts off because of sort of lack of knowledge’. (ID108F)
		Attitudes: ‘it's important to be heard because everybody has got a voice, and everybody is a key player in this…’. (ID104F)
		Attitudes: ‘ enlightening’, ‘it [CoVE] enhanced my views of why it's [vaccination] so important even more’. (ID128F)
		Knowledge: ‘… our fear on this side because we don't see it [the virus], we don't really understand it … That barrier is almost removed after, after being able to view or read through the package because it, it talks about the effectiveness, the dosage, the variants…’. (ID104F)
		Knowledge: ‘… It's education and getting more knowledge about this. And also promoting the [vaccination] service as well…’. (ID103F)
		Knowledge: ‘it was a bit of a refresher. You become a bit conscious of things that you can do to protect yourself and your loved ones and others you know’. (ID100F)
		Knowledge: ‘… it helps with my general understanding of COVID-19 and like knowledge increase’. (ID119F)
		Knowledge: ‘I think for, clinical-knowledge-wise or understanding the vaccine side effects or, or product delivery or manufacture will be useful for those who don't know’. (ID113M)
		Knowledge: ‘… it's quite a drastic change in my [knowledge] grading actually I think I graded it 3 out of 10 and then by the end of it was like 9 out of 10’. (ID124F)
		Knowledge: ‘extra knowledge in terms of COVID … understand the clinical side of things … how it's benefiting people that are breastfeeding and pregnant women … allowing me to kind of look at the extra resources to … go into a little bit more in detail about certain aspects of the resource, so these are probably what I’ll probably say that I've kind of learned a lot more about’. (ID124F)
		Knowledge: ‘… there's a lot of sort of niche questions that we don't have the answer to on top of our head, like for example, the COVID vaccine with pregnancy and breastfeeding. So this is sort of opened my eyes to a lot of the smaller questions that might not be relevant to all of the public, but a bit more specific to some certain patients’. (ID107F)
		Knowledge/Skills: ‘It was mostly just sort of, helped me to sort of enhance my knowledge on the COVID vaccine … I was able to talk to my parents about it, just sort of even just give them some information as well … I think I can give more information than I was able to beforehand, like before using the resource, I think now I'm equipped with a bit more information’. (ID118F)
		Skills: ‘I think I'm equipped to talk to anyone’. (ID106F)
		Confidence: ‘… [More confident in answering questions] especially in terms of like the, the vulnerable, … in our community, the older people, the people with chronic diseases’. (ID125F)
		Confidence: ‘… I feel like it does increase my confidence when I'm communicating to patients prior to administration’. (ID123F)
		Confidence: ‘So I think I feel a bit more confident with telling people about vaccines and why it's more useful than harmful. I think that's one thing, because I've always struggled with that with obviously there are loads of scares and, and the media and things’. (ID120F)
		Confidence/Knowledge: ‘Improving my confidence, improving my knowledge and just making me more aware of, you know the need to kind of share this information with people that might benefit’. (ID127F)
		Confidence/Knowledge: ‘… it has made me more confident about the knowledge, you know, about the vaccine and about it's, you know, it's importance, which I feel like, now I can, you know, even if I wasn't to use this, now I got this knowledge to kind of tell that to the patients. … it's improved my confidence about … the COVID vaccine and its benefits and things about it in more detail’. (ID128F)
		Confidence/Knowledge: ‘… I think because the statistics reassured me of my facts and my knowledge it has made me more confident …’ (ID122F)
		Commitment (to share learning): ‘ I will definitely pass it on to my family and friends. So, I think it is a benefit within itself’. (ID112F)
		Commitment (to share learning): ‘just making me more aware of, you know the need to kind of share this information with people that might benefit …’ (ID128F)
		Commitment (to share learning): ‘ The, the benefit will be because I’m in, in the, in contact with people in the health, health sector at the hospitals, but at the same time I'm a member of the community. So yeah, and a family member to my family. So yeah, a few other people were in the same position that I am [from EM community], I suppose word would spread around a bit quicker’. (ID111F)
Level 3: Behaviour	Application of learning: Changes to vaccine promotion behaviour OVP: occupational vaccine programme or peer communication CVP: community vaccine programme or patient communication Application of learning: Changes to protective behaviours (self, family, colleagues, wider community) Application of learning: Sharing knowledge with others (EMHCW, other colleagues, EM communities: family/friends, public/any knowledge recipient)	Vaccine communication (OVP): ‘it can convert people who are … non-COVID vaccine category and they don't wanna know, to actually after reading this, I think I've been convinced to give-it-a-go situation. So, you really are taking people out of the ‘I don't want to be vaccinated’ into ‘actually I'll give it a go’ place’. (ID104F)
		Vaccine communication (OVP): ‘… no one's actually taken [the vaccine] … they said they would still think about it and sort of just think if it's like the right move for them, but they feel like … I've given them a bit more information, a bit more insight to it’. (ID118F)
		Vaccine communication (OVP): ‘… It has definitely helped me signpost accordingly, especially with the menu being very, very clearly laid out. It has allowed me to, you know, quickly direct myself to a particular section …’ (ID117M)
		Vaccine communication (CVP): ‘Yes, when I will do any COVID clinics, the knowledge will, is obviously always applied for every patient’. (ID113M)
		Vaccine communication (CVP): ‘…as a vaccinator I have to obviously share knowledge with the patients and make sure they're well informed before I gain their consent to vaccinate. So, I have definitely used some of the information from this resource pack’. (ID108F)
		Vaccine communication (CVP): ‘Yeah, as a vaccinator recently there was a patient that did come in asking questions about clinical trials and you know how well it's been researched and it was useful to have the resource, how it broke it down into simple layman terms that I could relay back to the patient and the patient felt more put at ease. So that was good’. (ID129F)
		Hand hygiene/COVID-19 testing: ‘… I probably test myself and my family if we got symptoms and we're not sure if it's COVID and we probably tend to overdo the hand washing a bit and we have sanitiser in our cars as well. So maybe that's the only different thing that we started doing cause we stopped doing it … just being a bit careful when we are out in crowded places and so forth’. (ID100F)
		Face coverings: ‘Well, at work, we're still wear(ing) masks and we still take those measures, but it's, well after reading this it makes me realise that OK in other aspects of life, like when I go to uni or like see friends and families … we … should take these measures because we [are] still … in the middle of a pandemic. So at work we … follow those rules but outside we kind, we’re kind of lenient’. (ID114F)
		EMHCW/other colleagues: ‘… the staff members I work with, I did actually speak to them about this and thought it was really helpful …’ (ID115F)
		EMHCW/other colleagues: ‘So there, there are couple of … qualified doctors they've been asking me or show me also how are the vaccine developed. So they, they wanted to see and to read through also…. they were very interested to know that and I, I was sitting with them and was going with those quiz, that short quiz you have sent … so we all go through with that quiz so some of them, even people think that's true. They made it false and those false, they made it true. So yeah, it's, it's quite interesting…!’ (ID105F)
		EMHCW/other colleagues: ‘… like a qualified medical professional doctors also they have been listening to the videos and then enjoy the share using the quiz and things’. (ID105F)
		EMHCW/other colleagues: ‘I think the main benefit is just having the knowledge from reading it and then the capacity to share among my peers’. (ID121M)
		EMHCW/other colleagues: ‘I mentioned it to my colleague [name] but have not shared’. (ID102F)
		EM communities (family): ‘ it's really good to share with even my family members because it's easy for them to understand. It's got no like, doesn't have like big medical terms’. (ID114F)
		EM communities (family): ‘…the one time I shared it with my sister, she was curious, and she was actually going through stuff, and we were discussing things that she didn't know. She's obviously come from a different place where these things don't get, you know, shared. And for her, it was a bit of an eye opener. And she was quite, quite glad to see it because it made her a bit conscious of the fact that there were other things that she could do actually to protect herself and to protect the patients that she works with .… I think the resource helped her to almost signpost herself … We were discussing it and I was showing her and then I sent it to her. So, she was the one that was telling me that, oh I think I need to book myself with my GP to get my vaccines up to date, you know’. (ID100F)
		Any knowledge recipient: ‘… the benefit is of spreading knowledge to family, friends, loved ones, clients, patients. So, I think that's the best benefit because of the end of the day, knowledge is power and people will be well equipped if they know exactly what to do’. (ID106F)
		Any knowledge recipient: ‘it would be like first thing that would pop on, pop up on my head if, if someone had a question. So, I can basically send this to them for example’. (ID115F)
Level 4: Results	Reducing vaccine hesitancy and encouraging vaccine uptake	Vaccine uptake (self): ‘… I myself … I've got the, I got the vaccine. I got the COVID vaccine and I abide to the rules of being safe. And if ever there could be a situation where we need to like protect ourselves, like maybe the extra boosters or anything other that would come up in the future…’. (ID106F)
		Vaccine uptake (EMHCW/other colleagues)
		‘I would say… if you ask one to 10 degree I would say is yeah, 9 to 10 degree is achieved because she, she decided to [vaccinate] … while she was reading this resource or watching videos … she got the proper information’. (ID105F)
		Vaccine uptake/reducing hesitancy ( EMHCW/other colleagues): ‘… sharing my experience with people, to even motivate them to go for the vaccine, you see. Even people who were reluctant way before I read the package and also just after the pandemic’. (ID101F)
		Vaccine uptake (EM communities: family): ‘And she's [sister] going to have all the vaccines so that she's up to speed…’. (ID100F)
		Vaccine uptake (patients): ‘Yes [someone has changed their mind to have a vaccine after using the resources]. Yes, the one really good thing about this resource is they have a lot of extra resources added on …patients have come up with these questions and even if I don't really know the answer, directing them towards these extra resources has been useful’. (ID116F)
		Vaccine uptake (patients): ‘… it was that patient who came with queries about clinical trials and just general information and they then, after me saying all this information that I learned from the resource, they were more put at ease [patient changed their mind and chose to vaccinate]. So, I think it did matter for them’. (ID129F)

*Note*. General recipient: any recipient of knowledge communication (e.g., peer, family, friend and public).

Abbreviations: EMHCW, ethnic minority healthcare workers; HCW, healthcare workers; EM, ethnic minority; Other colleagues, workers in health and care services.

## Discussion

We found that CoVE was viewed to be high-quality, accessible, engaging, and relevant to EMHCW with applicability to the promotion of COVID-19 vaccines and booster vaccines.

CoVE was viewed to be a valuable contributor to addressing the key influencers of vaccine hesitancy in themselves and others. Participants had trust in the evidence-based materials, which equipped them with the awareness, knowledge, and confidence to recommend vaccination or have conversations with hesitant others, to explore their reasons for vaccine hesitancy, dispel myths, and improve vaccine literacy. For those participants reporting less knowledge about vaccines at the outset, CoVE was perceived to increase their preparedness for vaccine promotion practice, and for many of our participants, led to behaviour changes (ie, relating to health communication) that supported their vaccine promotion practice. All participants were committed to sharing their learning with others, and many had already done so within the short timeframe of the study. This is an important outcome given the known influence that HCW have over other people's vaccination decisions^10^ and the potential, therefore, for EMHCW to influence vaccination decisions of other EMHCW. Many participants gave specific examples of *how* CoVE had impacted their health communications by addressing existing knowledge gaps relating to vaccine development and testing and safety of vaccines for specific groups.

Participants in our study reported behaviour changes (related to personal engagement in vaccination and/or protective behaviours, implementing their learning through engagement in health communication with others, and/or sharing their learning with colleagues and wider ethnic minority communities). Despite a short time between training and interview, there were some reports of vaccination uptake in EMHCW or recipients of their health advice. Prior research shows that CoVE improves knowledge and confidence for promoting the COVID-19 vaccine in HCW in the UK and internationally.^24^ Our study builds directly on this prior work with findings specific to EMHCW, a population in which vaccine uptake in UK occupational health vaccination programs is particularly low,^13^ with high vaccine hesitancy observed in UK ethnic minority communities more widely.^2^

In the UK, although vaccination is generally not mandatory, it is strongly recommended that all employers, including those in health and social care, advocate the professional responsibility of vaccination and make efforts to ensure that their employees (including volunteers) are fully vaccinated unless they are exempt.^32^ The Chartered Institute of Personnel and Development (CIPD)^33^ reminds us that employers in the UK have justification to encourage their employees to be vaccinated, not least because they are obliged by The Health and Safety at Work Act (1974) to take reasonable steps to reduce workplace risks, but also because COVID-19 is a reportable disease under the Reporting of Injuries, Diseases and Dangerous Occurrences Regulations (RIDDOR).^34^ In terms of the vaccination policy, employers in the UK should outline their stance on vaccination, but take a voluntary approach which aims to “build trust and encourage employees to appreciate the benefits for themselves and others”.^32^ The UK government provides recommendations for all employers to help them support the vaccination of their workforce. These include (i) sharing information on the facts around vaccination (including the importance of COVID-19 vaccination), (ii) showing support for vaccination from senior leadership, (iii) engaging expert and community leaders, and (iv) providing support for employees to facilitate workforce vaccination.^32^

Vaccination policy is key to presenting the benefits of vaccination, and the public health contribution that employees can make by protecting themselves, their colleagues, and the wider community by vaccinating. CoVE training supports government recommendations through information sharing. This training could therefore be used to support the implementation of vaccination policy (and occupational health vaccination programs) in healthcare organisations as part of a workforce education program, which would raise awareness about vaccination, present the facts, and demonstrate employer encouragement for vaccinating. CoVE was developed in collaboration with experts and provides a persuasive case for vaccines and booster vaccines, providing consistent, accessible, and factual data, and counteracting misinformation. Since CoVE is an open access, free digital training resource, with low resource implications and flexibility of use (ie, can be accessed at a time and place to suit the end-user which is known to be effective^35^ and offers wide geographical reach), we recommend that healthcare employers provide CoVE to all HCW alongside occupational health invitations to vaccinate. This could support the rollout of occupational vaccine and booster vaccine programs, recognising that CoVE content would need to be periodically updated to reflect the changing landscape of vaccines. Signposting all employees within the organisation to CoVE might be facilitated by line managers and senior leaders, and/or community leaders who may be best placed to reach communities in which vaccine uptake is low (eg, engaging EMHCW staff network leaders to reach EMHCW). Nonetheless, as raised in our sample, workforce education related to vaccination should be coupled with opportunities for employers to listen to any concerns that HCW might have and engage in 2-way conversations with their employees to support decision-making.

This study has several strengths and limitations. We experienced some recruitment challenges in this study that were associated with two influential factors. First, the study coincided with a third year of the COVID-19 pandemic and its impact on health and care services, coupled with severe staffing shortages, competing demands and pressures for HCW during the recruitment period^36^ and an ongoing period of HCW strikes, nationally.^37^ Second, there are known barriers to research participation in ethnic minorities, where an underlying mistrust is proposed to hinder research engagement.^38^ Our original plan was to adopt a maximum variation sampling strategy, with strategic selection of individuals guided by age, gender, occupation, and seniority. However, due to the challenges experienced, we adopted a multidimensional approach to recruitment including snowball sampling, which took considerable time and resources, but significantly helped with recruitment. This aligns with prior research showing that snowball sampling can be effective for the recruitment of members of traditionally underserved populations.^39^ Further, knowing the community and cultural background of the target sample is recognised to be important in research recruitment with ethnic minorities.^40^ As such, our recruiting researchers were from ethnic minority backgrounds. Our recruitment suggests that under-served populations may be more likely to engage with in-person recruitment, particularly when approached by someone from a similar cultural or community group. This is supported by findings from clinical studies highlighting that face-to-face recruitment of individuals from ethnic minority backgrounds, when conducted by a researcher from a similar ethnic background, may increase the likelihood of recruiting participants from such backgrounds.^41^ Although our recruitment was mostly achieved online, we had more immediate success with face-to-face recruitment, and this personal contact may have helped to clarify the purpose of the study more effectively and establish participant-researcher trust.

Overall, our final sample size of 30 participants provided sufficient data to address our research questions and was larger than the qualitative sample [n = 15] in the original qualitative evaluation of CoVE with HCW, which adopted a similar framework approach.^24^ While we achieved some level of variation in age, gender, occupation, and seniority among our sample, many of our participants were women, and two-thirds were from the 21 to 30 years age group. Nevertheless, the gender balance reflects the higher proportion of women working in healthcare internationally (70% across 50 countries^29^), the UK National Health Service (around 68% women) and in specific roles included in our sample, such as allied health professionals (around 70% women), nurses (around 89% women), and midwives (around 91% women). Although we know less about the views of older EMHCW and those with other gender identifications, our findings have value since prior population-based research suggests that vaccine hesitancy is higher in women and younger age groups.^4^ Participants in our study were English language speakers; therefore, we do not know whether there are challenges in using CoVE training for those with language barriers. However, health and care workers in the UK are expected to speak, read, write, and understand English to at least level B1 on the Common European Framework of Reference for Languages (CEFR) scale; therefore, CoVE should be widely accessible to these professions.

We did not collect information from participants about religious beliefs, which are known to influence vaccine hesitancy.^42^ However, CoVE training is designed to be suitable for HCW of any faith. The training includes signposting to resources produced by religious leaders and faith communities (eg, the InterFaith Network for the UK), which were accessed by our participants and supported them in correcting misinformation and reducing religion-related vaccine hesitancy.

Finally, the individuals who came forward for interview expressed pro-vaccination views. Therefore, a limitation of the study is that we are unable to represent the views of those who are more vaccine hesitant or anti-vaccination (albeit we recognise the risk of social desirability bias). Nonetheless, this study demonstrates that CoVE is perceived by EMHCW to increase their knowledge and confidence for vaccine promotion, supporting their communication with others relating to vaccination (ie, health promotion practice), with examples provided of subsequent influence on their own, and others’ vaccination decisions.

Due to limited resources, and a tight timeframe to allow our study findings to have real-world value in supporting the rollout of the COVID-19 vaccines and booster vaccines during the pandemic, we collected qualitative data only. We suggest that future research includes the collection of pre-post quantitative measures to determine objective changes in knowledge following exposure to the training. A randomised controlled trial would be needed to determine the ‘effectiveness’ of CoVE in increasing the vaccination uptake in EMHCW.

Future studies should carefully consider culturally congruent research recruitment strategies, advocating altruism towards community, and adequate benefits for research participation^38^ as this builds trust and promotes mutually beneficial relationships. Qualitative research could be undertaken with a range of stakeholders, such as employers, managers, staff network leaders, community leaders, occupational health specialists, and policymakers, to explore how CoVE might be embedded within occupational health policy and practice.

## Conclusions

Our study provides novel and valuable insights into the specific views of EMHCW towards COVID-19 Vaccine Education (CoVE), a digital evidence-based training intervention, which aims to increase HCW knowledge about the individual and societal benefits of COVID-19 vaccination. Findings suggest that the CoVE training could be used to support the vaccination policy in health and social care employment settings to facilitate uptake of the COVID-19 vaccines and booster vaccines in EMHCW (as well as broader EM communities), which could have knock-on effects for public health.

Personal vaccination decisions are influenced by many factors, but access to, and trust in, evidence-based information is paramount. By providing evidence-based information, addressing reasons for vaccine hesitancy, dispelling myths, and improving vaccine literacy, CoVE supported pro-vaccination attitudes, and increased EMHCW knowledge and confidence to engage in conversations about the vaccine with others. Similar findings were evident in a broader evaluation of CoVE with HCW.^24^ HCW are exposed to COVID-19 infection through their work, yet EMHCW may be at greater risk for adverse health outcomes and mortality, although there remains low uptake of vaccines, high vaccine hesitancy, and heightened stigma related to vaccines in ethnic minority communities. Efforts to increase uptake of EMHCW in occupational health vaccination programs are urgently needed to reduce health risks for EMHCW, and our study provides valuable evidence that EMHCW found CoVE to be accessible, engaging, and relevant.

Specifically, the resource helped EMHCW to better understand specific concerns held by some ethnic minority communities towards vaccines (eg, related to religious beliefs or trust in vaccine development and testing) and discuss these with individuals from ethnic minority communities equipped with increased evidence-based knowledge and confidence.

Our findings provide early indication that CoVE, through enhancing knowledge about vaccines and confidence in health communications relating to vaccines, has the potential to increase vaccination uptake in ethnic minority communities, including encouraging EMHCW to participate in occupational health vaccination programs in healthcare employment settings. Quantifying long-term outcomes relating to vaccination uptake is beyond the scope of this qualitative study. Nonetheless, our study findings are novel and have implications for supporting the rollout of occupational health vaccination programs in healthcare settings, and ultimately, public health.

## Supplemental Material

sj-docx-1-new-10.1177_10482911241273914 - Supplemental material for A Qualitative Study of the Views of Ethnic Minority Healthcare Workers Towards COVID-19 Vaccine Education (CoVE) to Support Vaccine Promotion and Uptake Supplemental material, sj-docx-1-new-10.1177_10482911241273914 for A Qualitative Study of the Views of Ethnic Minority Healthcare Workers Towards COVID-19 Vaccine Education (CoVE) to Support Vaccine Promotion and Uptake by Holly Blake, Vinishaa Premakumar, Abishaa Premakumar, Aaron Fecowycz, Sala Kamkosi Khulumula, Wendy Jones and Sarah Somerset in NEW SOLUTIONS: A Journal of Environmental and Occupational Health Policy

sj-docx-2-new-10.1177_10482911241273914 - Supplemental material for A Qualitative Study of the Views of Ethnic Minority Healthcare Workers Towards COVID-19 Vaccine Education (CoVE) to Support Vaccine Promotion and Uptake Supplemental material, sj-docx-2-new-10.1177_10482911241273914 for A Qualitative Study of the Views of Ethnic Minority Healthcare Workers Towards COVID-19 Vaccine Education (CoVE) to Support Vaccine Promotion and Uptake by Holly Blake, Vinishaa Premakumar, Abishaa Premakumar, Aaron Fecowycz, Sala Kamkosi Khulumula, Wendy Jones and Sarah Somerset in NEW SOLUTIONS: A Journal of Environmental and Occupational Health Policy
