# Supplementary material for: A Qualitative Study of the Views of Ethnic Minority Healthcare Workers Towards COVID-19 Vaccine Education (CoVE) to Support Vaccine Promotion and Uptake
Source: New Solut. 2024 Sep 17;34(3):198–212. doi: 10.1177/10482911241273914 (PMC11490061; doi:10.1177/10482911241273914)
Supplement: sj-docx-1-new-10.1177_10482911241273914 - Supplemental material for A Qualitative Study of the Views of Ethnic Minority Healthcare Workers Towards COVID-19 Vaccine Education (CoVE) to Support Vaccine Promotion and Uptake [file sj-docx-1-new-10.1177_10482911241273914.docx]

**Supplementary file 1. Interview Topic Gide**

**Level 1: Reaction**

*(satisfaction)* What is your overall view of this resource?

*(engagement)* When using the resource, what is your view of the interactive elements? (by this we mean: menu, narration adjustments (can turn on or off and change the speed), video clips, ‘i’ information boxes, click boxes, quiz, extra resources).

*(relevance)* How relevant is this resource to you? Or to others? Will you have the opportunity to use this information or apply it in your studies or job?

**Level 2: Learning**

*(Knowledge)* Did you learn anything new from using this resource?

*(Skill)* Did this resource equip you with useful knowledge about the COVID-19 vaccine?

*(Attitude)* Do you think the COVID-19 vaccine is important for individual and societal health? Have you changed any of your views after using this resource?

*(Confidence)* Do you feel more confident to talk to other people (patients or clients) about the importance of the COVID-19 vaccine after using this resource?

*(Commitment)* Are you likely to use any of this information in the future? If so, how? Do you intend to share the resource? Tell us more.

**Level 3: Behaviour**

*(Behaviour)* Have you applied any of the knowledge from this resource in your life, studies, or job? If so, what, and how? Have you done anything differently?

*(Required drivers)* How do you think this resource should be best used? Who should use it and how is it best to provide them with access to it?

**Level 4: Results**

*(Leading indicators)* Have there been any other benefits of using or sharing this resource?

If you have applied this knowledge (e.g. by talking to others about the COVID-19 vaccine), do you think it has made any difference? To what degree? (prompt: have you been able to answer people’s questions better, or signpost people better, or changed anyone’s mind about the importance of the vaccine? Has anyone indicated that they have taken a vaccine based on your communication with them after using this resource?
