# Supplementary material for: A Qualitative Study of the Views of Ethnic Minority Healthcare Workers Towards COVID-19 Vaccine Education (CoVE) to Support Vaccine Promotion and Uptake
Source: New Solut. 2024 Sep 17;34(3):198–212. doi: 10.1177/10482911241273914 (PMC11490061; doi:10.1177/10482911241273914)
Supplement: sj-docx-2-new-10.1177_10482911241273914 - Supplemental material for A Qualitative Study of the Views of Ethnic Minority Healthcare Workers Towards COVID-19 Vaccine Education (CoVE) to Support Vaccine Promotion and Uptake [file sj-docx-2-new-10.1177_10482911241273914.docx]

**Supplementary Table S2**. Analytic Framework.

| Level 1 Reaction | |
| --- | --- |
| Favorable | Any mention of general satisfaction with the resource. Any mention of the resource being useful, practical, informative. Any mention of resource being seen as valuable. |
| Engaging | How well an individual was able to interact with the resource. Discussions of ease of use, lay language, accessibility for different learning styles, knowledge testing (quiz), interactive menus, videos, etc. |
| Relevant | How relevant the resource was to the individual, their job role, their community position and within their circle of colleagues, family and friends. Reference to CoVE being useful as a reference or confirming existing knowledge. Any mention of the relevance of information for addressing barriers to vaccination. |
| Level 2 Learning | |
| Attitudes | Any mention of participant / EMHCW / community attitudes. This may be positive or negative towards the resource or COVID-19/vaccination. This may include recognition of vaccine hesitancy. |
| Knowledge | Any mention of knowledge acquisition (self or others) from using the resource. Learning something new or confirming existing understanding. |
| Skills | Any mention of skills to implement knowledge through vaccine promotion. |
| Confidence | Any mention of self-confidence in communicating with others about vaccines and addressing vaccine-related concerns, questions or vaccine hesitancy. |
| Commitment | Any mention of desire or plans to share the resource with others (work colleagues, friends, family, public). |
| Level 3 Behavior | |
| Behavior changes | Any mention of the application of learning. Including actual changes in vaccine promotion behaviour (through occupational vaccination programmes, public vaccination programmes, or broader communication). Actual changes in other protective behaviours (including self, family, colleagues, wider community). Actions to share knowledge with others (such as EMHCW, other colleagues, EM communities: family/friends, public/any other knowledge recipient) |
| Level 4 Results | |
| Target outcomes | Any mention of behaviour changes leading to reduction in vaccine hesitancy, or vaccination uptake (self or others). |

CoVE: Covid-19 Vaccine Education; EMHCW: ethnic minority healthcare workers; EM: ethnic minority.
